# Low-Temperature Synthesis and Characterization of Hybrid Heavy Metal Zero- Dimensional Cluster Compounds Containing Pt/Ir and Bi.

**DOI:** 10.1063/4.0001178

**Published:** 2025-10-27

**Authors:** Gayomi Kanchana Samarakoon Mudiyanselage, Logan Schilling, Sviatoslav Baranets

**Affiliations:** Louisiana State Univesity, Baton Rouge, LA, USA

**Keywords:** Cluster, Ionic Liquids, Unit Cell Parameters, DFT, HOMO–LUMO Gap

## Abstract

Three novel hybrid organic-inorganic cluster compounds comprising 5d heavy metals (M = Pt, Ir) and Bi were synthesized at 180 °C in ionic liquid (IL) 1-butyl-3-methylimidazolium tetrafluoroborate ([BMIm][BF4]), which served both as reactant and reaction medium. Stoichiometric reactions between the appropriate metal halide and [BMIm][BF4] yielded three distinct cluster-containing salts: [PtBi6Cl12][BMIm]2, [Pt2Bi12Br22][BMIm]2, and [IrBi6Cl12][BMIm]3, all crystallizing in three different structure types. [PtBi6Cl12][BMIm]2 crystallizes in the monoclinic space group P21/c with the unit cell parameters of a = 9.1154(5), b = 9.0692(4), c = 23.8159(13), and β = 93.890(2) while [IrBi6Cl12][BMIm]3 crystallizes in the monoclinic space group C2/c (a = 22.0181(7), b = 12.6726(4), c = 35.2412(11), β = 95.132(1)) both featuring cuboctahedral [MBi6Cl12]x– (x = 2, 3) anionic clusters. The Br-bearing analog [Pt2Bi12Br22][BMIm]2 crystallizes in the triclinic space group P (a = 9.3063(3), b = 12.4615(4), c = 14.7424(5), α =70.484 (10), β = 87.966(10), γ = 87.497(10)) and exhibits double cuboctahedra [PtBi12Br22]2– cluster. Each cluster anion is charge-balanced by [BMIm]+ ions, providing a unique combination of the lightest and heaviest elements in the title compounds. Molecular DFT calculations reveal that each anionic cluster, [PtBi6Cl12]2–, [IrBi6Cl12]3–, [PtBi12Br22]2– has HOMO–LUMO gaps of 3.3774 eV, 3.2332 eV and 2.4106 eV respectively. Details of the synthesis, crystal and electronic structures, and perspectives for semiconducting applications are discussed.

